# Combining Living Microorganisms with Regenerated Silk Provides Nanofibril-Based Thin Films with Heat-Responsive Wrinkled States for Smart Food Packaging

**DOI:** 10.3390/nano8070518

**Published:** 2018-07-11

**Authors:** Luca Valentini, Silvia Bittolo Bon, Nicola M. Pugno

**Affiliations:** 1 Dipartimento di Ingegneria Civile e Ambientale, Università di Perugia, UdR INSTM, Strada di Pentima 4, 05100 Terni, Italy; silvia.bittolo@gmail.com; 2 Laboratory of Bio-Inspired and Graphene Nanomechanics, Department of Civil, Environmental and Mechanical Engineering, University of Trento, I-38123 Trento, Italy; 3 School of Engineering and Materials Science, Queen Mary University of London, Mile End Road, London E1 4NS, UK; 4 Ket-Lab, Edoardo Amaldi Foundation, Italian Space Agency, via del Politecnico snc, I-00133 Roma, Italy

**Keywords:** bionic composites, thin films, mechanical properties

## Abstract

Regenerated silk (RS) is a protein-based “biopolymer” that enables the design of new materials; here, we called “bionic” the process of regenerated silk production by a fermentation-assisted method. Based on yeast’s fermentation, here we produced a living hybrid composite made of regenerated silk nanofibrils and a single-cell fungi, the *Saccharomyces cerevisiae* yeast extract, by fermentation of such microorganisms at room temperature in a dissolution bath of silkworm silk fibers. The fermentation-based processing enhances the beta-sheet content of the RS, corresponding to a reduction in water permeability and CO_2_ diffusion through RS/yeast thin films enabling the fabrication of a mechanically robust film that enhances food storage durability. Finally, a transfer print method, which consists of transferring RS and RS/yeast film layers onto a self-adherent paraffin substrate, was used for the realization of heat-responsive wrinkles by exploiting the high thermal expansion of the paraffin substrate that regulates the applied strain, resulting in a switchable coating morphology from the wrinkle-free state to a wrinkled state if the food temperature overcomes a designed threshold. We envision that such efficient and smart coatings can be applied for the realization of smart packaging that, through such a temperature-sensing mechanism, can be used to control food storage conditions.

## 1. Introduction

Living microorganisms have long been used in food preservation [1,2]; such microorganisms form living surfaces that provide an attractive platform for the development of functional materials. At present, biotech companies uses fungi to produce valuable products [3]; thus, combining the fermentation mechanism of some microorganisms with biomaterials could give rise to bionic composites with novel properties.

Among such novel products, the development of innovative packaging solutions to increase the shelf-life of fresh fruits by slowing down their metabolism so they remain fresh and appetizing for longer, and sensors to monitor if perishable food in the cold chain is maintained in the desired temperature range to prevent the growth of pathogens and spoilage microorganisms, remains a challenge. To date, biodegradable polymers (i.e., polylactic acid or polylactide (PLA), polyglycolic acid or polyglycolide (PGA), poly-caprolactone (PCL), and polyhydroxybutyrate (PHB)) have been explored for food packaging due to mechanical robustness of the matrix with hydrophobic chain groups that allow for low permeability to oxygen and water vapours [4]. However, their biocompatibility, lamination without solvent-based adhesives, and sensing properties are still challenging aspects for their utilization in smart packaging applications.

Concerning the sensing issue, the spontaneous generation of wrinkles induced by the buckling of a thin skin due to thermal contraction of the underlying substrate may be used as smart sensors for monitoring temperature changes in a food cold chain. In this regard, micro/nanoscale surface patterns obtained by coupling a stiff skin to a soft substrate have been used to create reversible patterns that are responsive to temperature and provide a unique surface morphology to sense temperature changes [5,6,7].

From the material point of view, silk fibroin is an ideal candidate for packaging applications since it is a biocompatible structural protein that can be processed to obtain films which recently have been used as biodegradable and edible sensors to monitor food degradation [8,9,10]. The polymorphism of silk fibroin (i.e., random coil, silk I, and silk II structures) can be also tailored by controlling the content of β-sheet crystals to enable the correct gas exchange and water vapour permeability through silk-based membranes [11,12,13]. Among the different fabrication methods, transfer printing is the most known method for interfacing silk on soft substrates [14]. In this regard, the hidden strength and stiffness of natural honeycomb walls constructed from recycled silk and wax secreted by worker bees [15,16,17] is reminiscent of modern fiber-reinforced composite laminates.

Taking inspiration from the honeybee comb cell wall, a self-adhesive and soft thermoplastic paraffin wax can be used to stick a regenerated silk (RS) film to produce a bilayer system [18]. Being the paraffin wax a material with a high thermal expansion coefficient, wrinkles occur to minimize the total energy of such a bilayer system when the compressive strain, caused by the thermal expansion coefficients and rigidities, mismatches between the skin layer and the substrate induced by thermal stimulus.

Inspired by our previous work where the metabolic activity of living microorganisms was used as an engineered platform for the fabrication of advanced carbon-based materials [19], here we extend this approach, with emphasis on the fermentation process used for centuries in wine- and bread-making, to produce bionic composites which integrate regenerated silk nanofibrils that from the geometrical point of view and in terms of mechanical properties are similar to carbon nanotubes. The resulting reduced volume fraction of nanofibrils within the film could make the fermented hybrid composite more resistant to fracture with self-repairing properties exploiting the microorganisms’ growth process that allows for the intracellular transport of nanomaterials, and the CO_2_ bubbles produced during fermentation could be used to produce porous architectures for biomedical applications. Here, it was observed that once *Saccharomyces cerevisiae* yeast cells were fermented by nutrient addition into a silk fibroin solution, the regenerated silk shows a higher content of beta-sheet structures. Moreover, the microorganism growth increased the cell density and reduced the porosity of the RS membrane, limiting the exchange of water and gas diffusion. As conceptual proof, we demonstrated as an example that the deposition of such a living coating on fruits helps the preservation of their shelf-life. Finally, we demonstrate that RS-based film layers can be laminated onto a paraffin wax substrate for the realization of temperature-responsive bilayer system.

## 2. Materials and Methods

For the preparation of the RS film, commercial *Bombyx mori* silk cocoons were boiled for 1 h in a distillated water solution of 0.025 wt % NaHCO_3_ rinsed with distilled water every 30 min to remove the sericin. According to the method adopted by Kaplan et al. [20], the degummed silk (i.e., 0.2 g) was then added to a CaCl_2_ (i.e., 0.14 g) and CH_2_O_2_ (formic acid) (i.e., 20 mL) solution mixture and stirred overnight at 40 °C to yield a 1 wt % solution. A water solution (50 mg/mL) of a *Saccharomyces cerevisiae* (Lesaffre Italia S.p.A. S. Quirico, Tre Casali, Italy)-based beer yeast extract was prepared separately by mechanical stirring at 30 °C for 1 h. After that, 0.4 g of sucrose was added to 20 cc of water to start the fermentation. The water solution of fermenting yeast was then added to the silk fibroin solution. RS/yeast films were prepared by leaving the silk–yeast solution to evaporate for 12 h in a polystyrene Petri dish (diameter 15 cm). The growth of yeast cells was monitored by the optical density (OD) method, measuring the absorbance at a wavelength of 600 nm and a temperature of 30 °C of the yeast and RS/yeast solutions in sucrose growth medium. The morphology of the films was investigated by optical and field emission scanning electron microscopy (FESEM). Fourier transform infrared (FTIR) analysis was performed in a Jasco FTIR FT/IR-615 spectrometer equipped with an ATR mode in the wave number range from 400 to 4000 cm^−1^. The spectra were deconvoluted by firstly smoothing the signal with a polynomial function with a 15-point Savitski–Golay smoothing function, subtracting a linear baseline, and applying a Gaussian deconvoluting curve by Origin 9 software. X-ray diffraction (XRD) was performed using a Bruker D8 Advance diffractometer with a radiation source of CuKα and wavelength λ = 0.154 nm operated at 40 kV and 40 mA. The incidence angle (2θ) was varied between 2° and 60° and the scan rate was 0.02°/s. The tensile properties of films were measured using a universal tensile testing machine (Lloyd Instr. LR30K) with a 50 N static load cell. Three specimens of each sample were cut into strips (30 mm × 12 mm × 0.08 mm). The gauge length was 20 mm, and the extension rate was set at 2 mm/min.

The effect of different types of coatings on bananas’ freshness was evaluated by monitoring the colour change through time-lapse photography. The water permeability was determined after soaking a sponge in water and subsequently dip-coating the sponge in RS and RS/yeast solutions. The variation of the weight was monitored at different hours with a standard laboratory balance (Mettler Toledo AB135-S/FACT). The weight variation was calculated as an average of three measurements for each coating. The respiration rate of bananas was evaluated by monitoring the CO_2_ production. In brief, bananas were placed in a sealed FTIR chamber and the production of CO_2_ was monitored by measuring the evolution of the CO_2_ absorption peak over a period of 7 days (see Supplementary Material Figure S1). This measurement takes into account the initial background performed in air to remove the initial contribution of the carbon dioxide moisture of the air.

For the adopted transfer print process to realize the bilayer system, regenerated silk was transferred to a Parafilm film (Parafilm M^®^, Bemis Company Inc., Neenah, WI, USA) through a direct transfer process, which consists of placing RS and RS/yeast free standing films on the receiving Parafilm substrate while applying with a hot press a pressure of 2 kPa at 60 °C for 15 min; the composite material separates spontaneously from the press plates as it cools. The obtained bilayer systems were heated at 60 °C and cooled to room temperature (i.e., ΔT ≈ 40 °C), and atomic force microscopy (AFM) was carried out to measure the wrinkle morphology using a Nanosurf easyScan DFM system in tapping mode with Gwyddion 2.51 free software for image processing.

## 3. Results and Discussion

**Figure 1:**
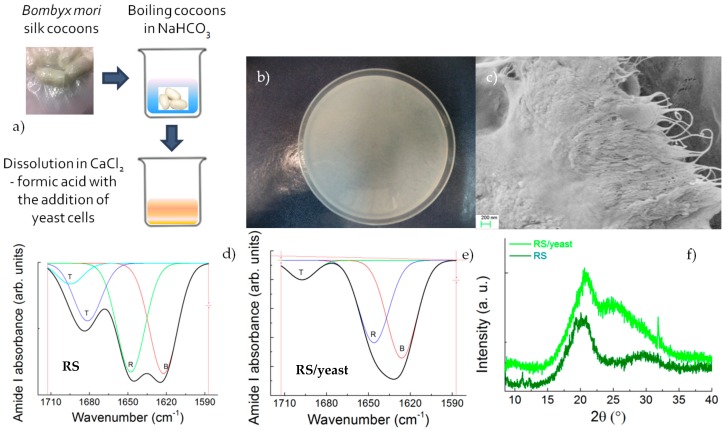
(**a**) Scheme of silk fibroin production using regenerated silk (RS); (**b**) Visual appearance of the RS/yeast film and (**c**) FESEM image of silk nanofibrils; (**d**,**e**) FTIR spectra of regenerated silk and RS/yeast films, respectively. The coloured lines represent the components of the amide I band and are indicated as β-sheets (B), random coils (R), and turns (T); (**f**) XRD results of regenerated silk and RS/yeast films.

The production method adopted for the realization of thin films with the aid of living microorganisms is schematically reported in Figure 1a. The yeast fermentation was implemented into a CaCl_2_–formic acid dissolution system, which can be used to produce large films (Figure 1b) with a nano-fibrillar structure (Figure 1c).

Changes in the structure of the films deposited after the fermentation-assisted silk dissolution were detected by FTIR and XRD. The β-sheet (crystalline) content was determined by the deconvolution of the amide I region (1580–1720 cm^−1^) and estimating the ratio between the peak area in the wavenumber region of 1622~1637 cm^−1^, which is the main absorbance region of β-sheet crystal in amide I [21], and the whole area of the amide I region comprising the peaks of the structural components, including turns (T) and random coils (R). The deconvolution of the amide I band provides an estimation of the β-sheet structure in the RS and RS/yeast of 37% and 44%, respectively (Figure 1d,e).

The XRD data in Figure 1f show that the RS/yeast film is characterized by diffraction peaks at 2Θ values of 20.4° and 25.4°, corresponding to a silk I structure [22,23]. The RS film also showed silk I and silk II crystal structures, having diffraction peaks at 20.4° and 29.0°. Compared with RS/yeast, the silk I peak at 25.0° disappeared, indicating more silk II formation.

**Figure 2:**
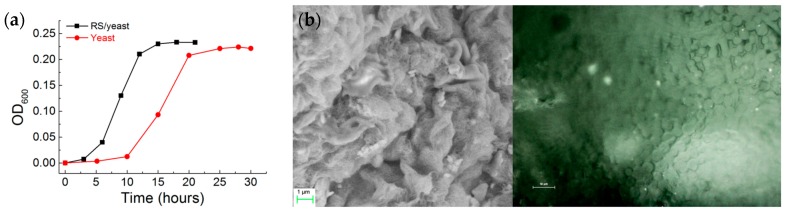
(**a**) Optical density (OD) measured at a 600-nm wavelength during growth of neat *Saccharomyces cerevisiae* and *Saccharomyces cerevisiae* in RS with sucrose until the stationary phase was reached; (**b**) Appearance under FESEM and optical microscope of the RS/yeast cell growth during the stationary phase.

The cell division of yeast occurs by budding in which a daughter cell is initiated from the mother cell followed by nuclear division and finally cell separation. The yeast cell growth reported in Figure 2 shows three main phases: a lag phase where the individual cells are activated in preparation for division; an exponential phase once the cell starts actively metabolizing shortly after the cells divide; and finally a stationary phase when the metabolism slows and the cells stop rapid cell division [24]. The factors that cause cells to enter the stationary phase are related to change in the environment typically caused by high cell density. The data reported in Figure 2a state the stability of the yeast cells to proliferate also with the presence of formic acid in the nutrient broth. The effects of RS addition during the growth curve are demonstrated in Figure 2a by measuring the OD during the cell growth. The OD curve of the yeast cells is substantially altered by the RS addition, with the effect on the lag time and final cell yield being particularly pronounced: RS/yeast cells have a lag phase of ~2–3 h, after which they proliferate rapidly; in comparison, the neat yeast cells show an increased lag phase of ~10 h. The morphology of the stationary phase for the RS/yeast system observed by means of FESEM and optical microscopy (Figure 2b,c) indicates the cell proliferation for such culture.

In order to demonstrate the potential application of such a living coating in consumer-exposed food, the mechanical robustness of the films is required to withstand, for example, handling procedures.

**Figure 3:**
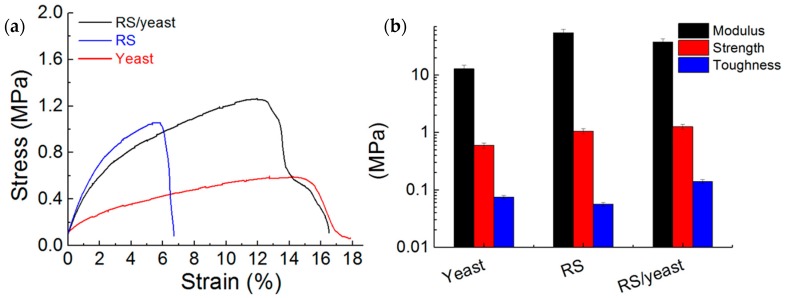
(**a**) Stress/strain curves from testing of yeast, RS, and RS/yeast samples. The curves represent an analysis performed on three different samples; (**b**) Effect of yeast fermentation on modulus, strength, and toughness of RS film. The modulus, strength, and toughness of neat yeast film have been reported for comparative purposes.

Figure 3a represents typical stress/strain curves obtained from testing of yeast, RS, and RS/yeast samples. The maximum average toughness (i.e., the area underlying the stress/strain curves) obtained from the RS/yeast sample was 0.14 MPa (Figure 3b), the highest average strength (i.e., the stress at the ultimate strain) obtained was 1.26 MPa, and the maximum elastic modulus recorded was 37.2 MPa. The improved mechanical properties with the fermentation-based dissolution of silkworm silk fibers agree with our previous studies reported for the fermentation-assisted synthesis of bionic composites [19,25]. In these studies, addition of carbon nanotubes (CNTs) and/or graphene in the fermentation broth resulted in composites with a higher toughness value. In our studies, tensile tests on dried composite films were rationalized in terms of a CNT cell-bridging mechanism where the strongly enhanced strength of the composite is governed by the adhesion energy between the bridging CNTs and the matrix. The presence of glucose can plasticize regenerated silks and increase the ultimate strain leading to toughness values (see Table 1) that are comparable to those measured for traditional biopolymers used for food packaging [26,27] being in our case as added value the edibility of the coating.

**Figure 4:**
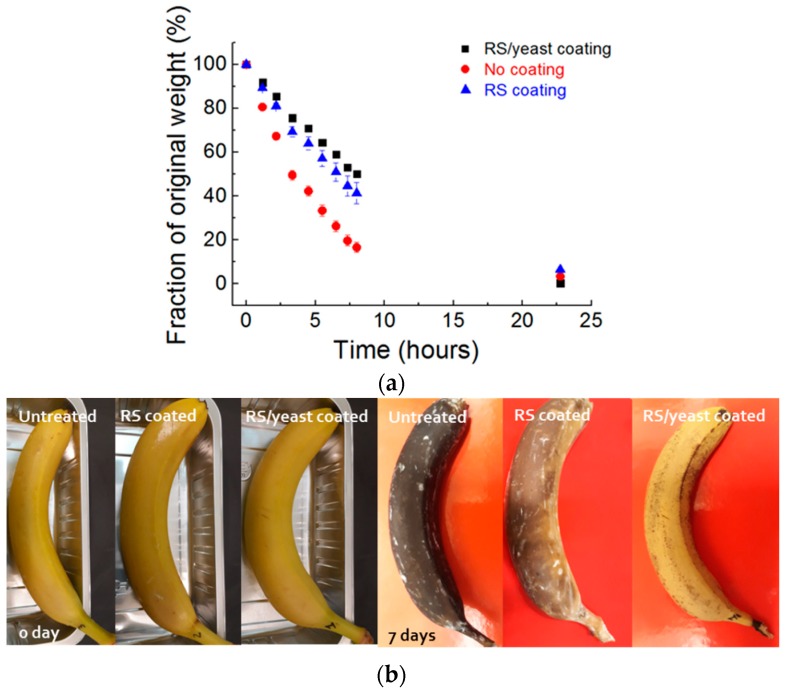

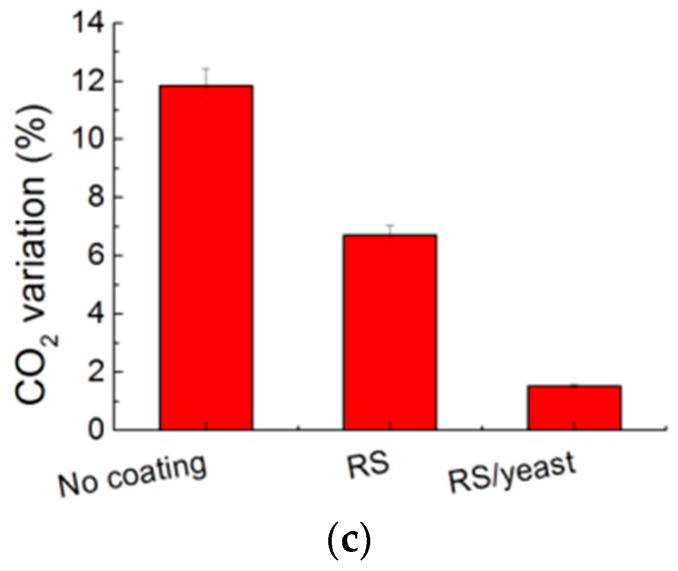
(**a**) Weight loss of sponges soaked in water and stored for up to 25 h at 23 °C and 32% relative humidity (RH). Sponges were stored without coating or after dip coating with RS and RS/yeast suspensions, respectively; (**b**) Evaluation of bananas’ degradation. Fruits were stored at 23 °C and 32% RH as received and after coating with a silk/yeast film. Time lapse photography of banana degradation, indicating that the silk/yeast coating reduces the degradation rate; (**c**) CO_2_ variation of uncoated, RS-coated, and RS/yeast-coated bananas, respectively.

In general, coatings for food packaging beyond the mechanical robustness should exert low permeability to water vapors; fruit dehydration is, in general, an indicator of the breakdown of the protective skin, which results in loss of turgor and water evaporation. We observe that the increase in beta-sheet content yields a lower water permeability through the RS/yeast membrane as indicated by the lower variation in the initial weight of soaked sponges coated with different types of coatings as reported in Figure 4a (see Supplementary Material Figure S2). These results are in agreement with those obtained by Omenetto et al. [10], who showed that when the silk fibroin beta-sheet content is in the range of 36–58%, the water vapour permeability is 5 times smaller than that measured for the film with a lower beta-sheet content.

Many fresh fruits have high metabolic activity, and due to microbial attack it results in a short conservation time, colour change (Figure 4b), and off-flavour. The change in colour of fresh fruit, in particular, is associated with ethylene production and cell respiration. To evaluate the exploitation of RS/yeast film as a barrier coating, the change in color of coated and non-coated fresh bananas was evaluated (Figure 4b). Time-lapse photography shows that an RS/yeast coating decreases the fruit degradation when compared to uncoated or RS-coated fruit at day 7. During the continuing metabolism of the fresh fruit, oxygen is transformed into carbon dioxide; thus, gas permeation through a coating film plays a crucial role in fruit storage. To prevent the spoilage of fresh fruits, it is necessary to reduce the breathing process. In our case, the higher beta-sheet content of the RS/yeast film decreases the production of CO_2_, which indicated a decrease in the respiration rate of the fruits (Figure 4c).

**Figure 5:**
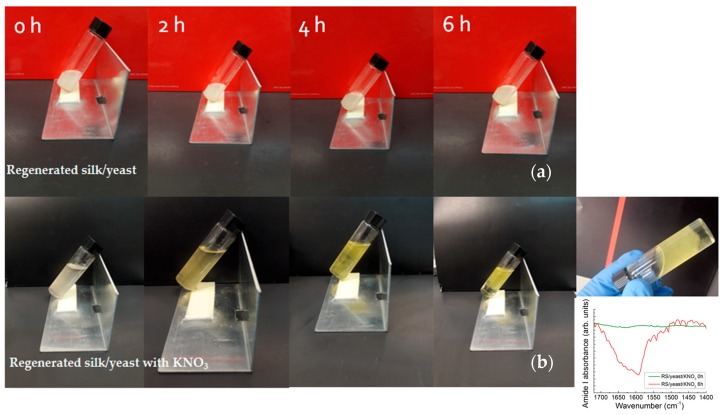
Dynamic optical morphology of (**a**) RS/yeast and (**b**) RS/yeast/KNO_3_ solutions resulting at different times (temperature 37 °C). The inset of panel (**b**) shows the evolution of the amide I FTIR band of the RS/yeast/KNO_3_ solution with time and gelation of the RS/yeast/KNO_3_ solution.

Another very interesting property of such silk nanofibrils relies on their ability to self-assemble, giving rise to a sol–gel transition with rapid gelation time induced by the presence of salts [28,29]. Pregelation occurs when a fresh solution has a β-sheet content of about 20% with negligible intermolecular bindings; gelation is then induced by interchain interactions that become irreversible with the formation of the β-sheet intermolecular structures of the gel phase [29]. In our case, the gelation was observed when KNO_3_ salt was added to a silk nanofibrils/yeast/formic acid solution (Figure 5). Without salt, the RS/yeast retains with time a sol characteristic (Figure 5a). In comparison to the sol RS/yeast solution, the RS/yeast with salt solution transforms into a semi-solid gel within several hours together with the appearance of a strong infrared absorption peak at 1626 cm^−1^ due to the formation of strong β-sheet structures (Figure 5b).

**Figure 6:**
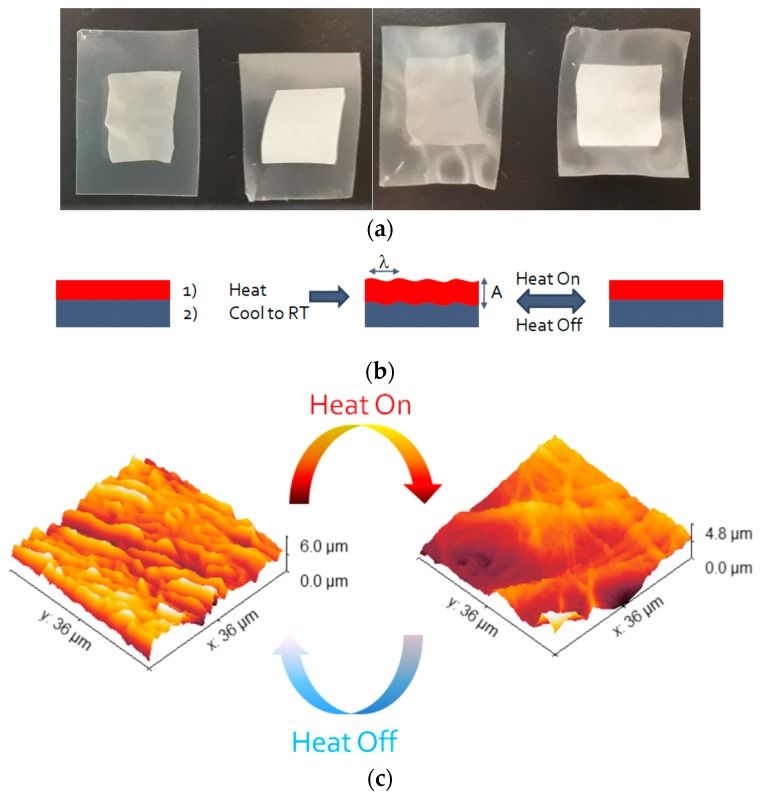

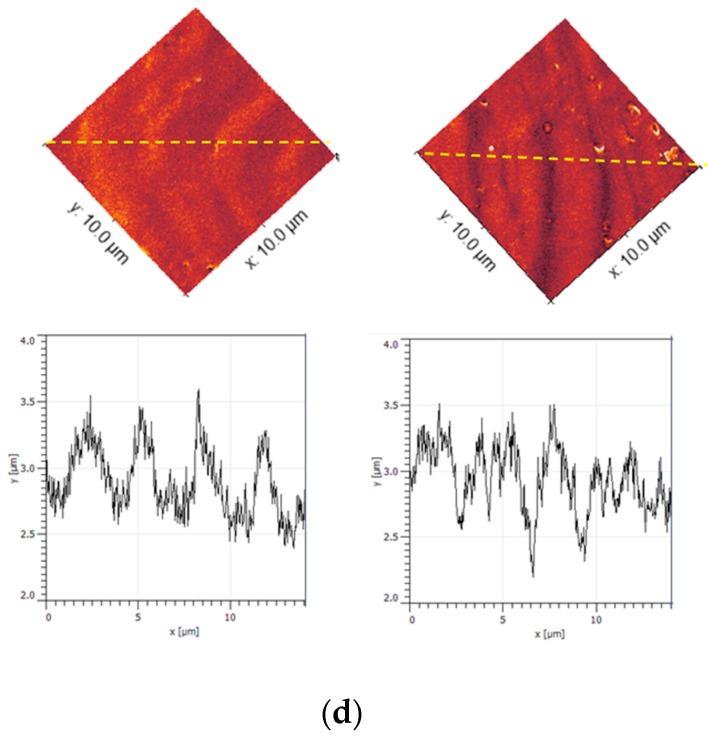
(**a**) Photographs of RS/yeast/paraffin and RS/paraffin bilayer systems before (left) and after (right) the wrinkle activation; (**b**) Schematic illustration of temperature-driven wrinkling. The bilayer system is flat at room temperature and the heat induces the temperature to increase; thus, the thermal expansion of the paraffin substrate results in an increase in the compressive strain of the bilayer systems once they have cooled down to room temperature with the appearance of the wrinkles. Once a wrinkled state is activated, the heat/temperature can again induce thermal expansion of the paraffin substrate, resulting in the reversibility of the temperature-driven wrinkles; (**c**) three-dimensional (3D) AFM images showing the reversibility of the temperature-driven wrinkles; (**d**) AFM images and the related profile of the yellow lines passing through the RS (left) and RS/yeast (right) film layers.

Considering the application of controlling the food cold chain, it is essential to design a bilayer packaging system where the mechanical properties of the top skin layer when laminated onto a soft substrate will be beneficial for the creation of temperature-driven surface patterning. Figure 6a shows the RS and RS/yeast films laminated onto a paraffin wax substrate. Wrinkle formation is typically connected to the high thermal expansion coefficient of the substrate used for the transfer. Parafilm is used worldwide in research laboratories as a self-adhesive and sealant plastic foil. It is soft (tensile strength ≈2.0 MPa) with a high thermal expansion coefficient (i.e., 0.89 × 10^−3^ K^−1^) and due to its low melting point (≈60 °C), it becomes adhesive when applying heat during lamination and sticks strongly to the receiving material. The formation of wrinkles occurs to minimize the total potential energy of the skin layer and the substrate induced by thermal expansion. The strategy for the realization of heat-driven wrinkle patterns is illustrated in Figure 6b; once heated, the paraffin wax upon cooling to room temperature generates a compressive stress at the interface of the bilayer sample owing to the considerable mismatch between the modulus and thermal expansion ratio of the substrate and the stiff top layer made of RS or RS/yeast [30]. The wrinkling formation and height analysis of wrinkles can be determined with the use of the AFM. The AFM images reported in Figure 6c show that the crumpled surface appears unfolded and smoothed once heated. Height histograms spanning a few wrinkles of the RS and RS/yeast cooled samples are given in Figure 6d and indicate that the step heights are ≈1 μm high. The highest peak distributions indicate the periodicity in the surface morphology. Furthermore, it can be easily noticed that film layers form long line-shaped wrinkles (length in the order of 2 μm). Moreover, it is clearly evidenced by AFM height histograms that the film layer between two successive wrinkles remains at the same height level, suggesting that the film is now lying upon its substrate (Figure 6d).

Further analysis of the AFM images reveals that the size of the peak (wrinkles’ wavelength) distribution decreased in the RS/yeast sample, indicating that, among the samples studied, the stiffer film layer produced the smallest periods and amplitudes. The periods of the wrinkle structures were measured by using Gwyddion 2.51 free software, and the amplitude (A) and wavelength (λ) of the wrinkled structures are reported in Table 2.

The amplitude and the wavelength of the wrinkles depend on the thickness and mechanical properties of the skin layer and the substrate according to linear bucking theory [31,32,33,34,35], which can be expressed as:

A = h_f_((σ_0_ − σ_c_)/σ_c_)^1/2^/a (1)

and

λ = 4.36h_f_(E_f_(1 − ν_s_^2^)/(E_s_(1 − ν_f_^2^))^1/3^ − 2A(1 − a)/a (2)

where σ_c_ refers to the critical stress to wrinkle formation and is given as^33^

σ_c_ = (1/4)(E_f_′)^1/3^(E_s_′)^2/3^ (3)

and σ_0_ is the compressive stress of the film layer at a temperature below the heating temperature, which is given by the equation [36]:

σ_0_ = (E_f_(α_s_ − α_f_)ΔT)/(1 − ν_f_) (4)

where E′ = E/(1 − ν^2^) and the subscripts f and s refer to the film layer and the substrate of the bilayer system, respectively, and E′, E, and ν are the plane modulus, Young’s modulus, and Poisson’s ratio, respectively. *h*_f_ is the film thickness and 0 ≤ a ≤ 1 is an adhesion parameter that we have introduced (ideally a = 1) for accounting for the non-ideal bonding between film and substrate (imposing the film’s inextensibility and simply assuming squared-shape wrinkles, i.e., 2A + λ cost). The Young’s modulus for RS, RS/yeast, and paraffin are 54 MPa, 37 MPa, and 1.4 MPa [18], respectively, and ν_f_ = 0.5. The applied strain ε when the bilayer system is heated is calculated as ε = (α_s_ − α_f_)xΔT, where α_s_ and α_f_ are the thermal expansion coefficients with α_s_ >> α_f_. Finally, the theoretical wavelength and amplitude values obtained from Equations (1) and (2) are reported in Table 2 and compared with the experimental findings by fitting the single parameter a.

## 4. Conclusions

Here, we described how the activation of the metabolic activity of microorganisms with regenerated silk increases the crystalline content of RS fibroin, reducing the water permeability by ≈30% and increasing the shelf-life of perishable food over a period of 7 days. Then, we reported a method which consists of transfer printing the prepared freestanding RS and RS/yeast layer films onto a self-adherent Parafilm substrate. By increasing the applied strain (ε) via thermal heating of the high thermal expansion paraffin-based substrate, we observed an increase of the wrinkles’ wavenumbers in the RS/yeast skin layer due to a high mechanical mismatch between the film layer and the paraffin substrate. This approach can be used in the real design of smart coatings by controlling the mechanical properties as the designing parameter for tuning the wrinkle morphologies. Moreover, the proposed method for coupling two different systems could be considered as a valid alternative for laminating two or more flexible layers without a bonding agent. Such solventless laminates could be much more environmentally friendly and result in a low-cost product for high-speed production lines.

## Figures and Tables

**Table 1:** Mechanical properties of various biopolymers for food packaging [27]. σ*, specific tensile strength; E*, specific tensile modulus; ε, ultimate strain.

Type of Biopolymer	Toughness (MPa)	σ* (Nm/g)	E* (kNm/g)	E (%)
RS/yeast (this work)	0.14	1.1	0.032	15.4
PLA	0.26	16.8	0.3	2.5
PLLA	0.23	40.0	2.2	3.0
PDLLA	0.28	22.1	0.8	2.0
PDLLA/PGA50/50	0.41	30.9	0.8	2.0

**Table 2:** Theoretical and experimental (estimated by AFM images and related profiles reported in Figure 6d) amplitude and wavelength of the different skin layers with a = 0.4.

Sample	A_Theor._ (μm)	λ_Theor._ (μm)	A_Exp._ (μm)	λ_Exp._ (μm)
RS/paraffin	2.72	1.83	1.23	2.93
RS/yeast/paraffin	2.20	2.21	1.04	2.14

## Supplementary Materials

The following are available online at http://www.mdpi.com/2079-4991/8/7/518/s1, Figure S1: Evolution of the CO_2_ absorption peak over period of 7 days for (left) RS/yeast and (right) RS coatings, respectively; Figure S2: Water-soaked sponges with different types of coatings.
