# Supplementary material for: Combining Living Microorganisms with Regenerated Silk Provides Nanofibril-Based Thin Films with Heat-Responsive Wrinkled States for Smart Food Packaging
Source: Nanomaterials (Basel). 2018 Jul 11;8(7):518. doi: 10.3390/nano8070518 (PMC6071141; doi:10.3390/nano8070518)
Supplement: Supplementary file 1 [file nanomaterials-08-00518-s001.pdf]

Supplementary Materials

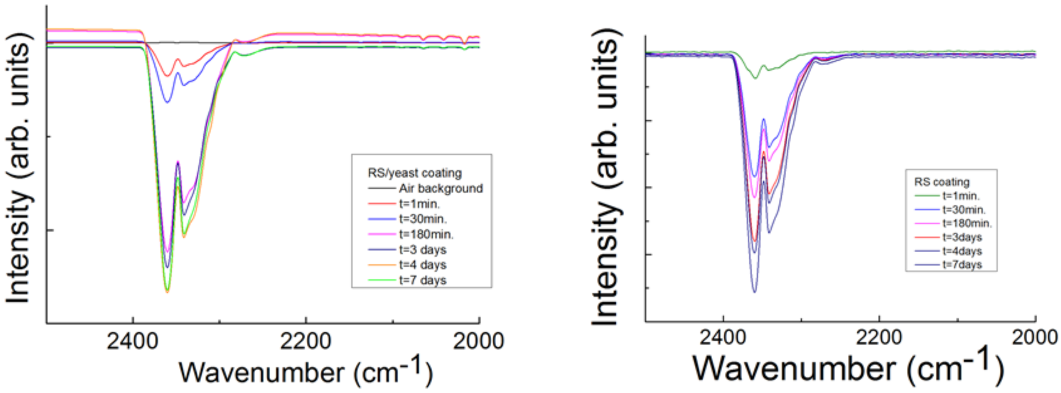

Figure S1. Evolution of the CO_2 absorption peak over period of 7 days for (left) RS/yeast and (right) RS coatings, respectively.

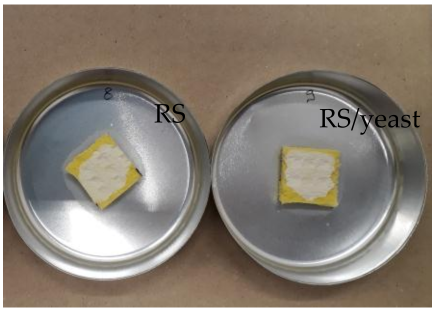

Figure S2. Water soaked sponges with different types of coatings. On the left it is evident the drying region around the sponge due to the water loss.
